# Extracting rate changes in transcriptional regulation from MEDLINE abstracts

**DOI:** 10.1186/1471-2105-15-S2-S4

**Published:** 2014-01-24

**Authors:** Wenting Liu, Kui Miao, Guangxia Li, Kuiyu Chang, Jie Zheng, Jagath C Rajapakse

**Affiliations:** 1 School of Computer Engineering, Nanyang Technological University, Singapore, Singapore; 2 Genome Institute of Singapore, A*STAR (Agency for Science, Technology and Research), Singapore, Singapore

**Keywords:** gene regulatory network (GRN), time delays, transcriptional regulation, rate changes, gene regulation ontology

## Abstract

**Background:**

Time delays are important factors that are often neglected in gene regulatory network (GRN) inference models. Validating time delays from knowledge bases is a challenge since the vast majority of biological databases do not record temporal information of gene regulations. Biological knowledge and facts on gene regulations are typically extracted from bio-literature with specialized methods that depend on the regulation task. In this paper, we mine evidences for time delays related to the transcriptional regulation of yeast from the PubMed abstracts.

**Results:**

Since the vast majority of abstracts lack quantitative time information, we can only collect qualitative evidences of time delays. Specifically, the speed-up or delay in transcriptional regulation rate can provide evidences for time delays (shorter or longer) in GRN. Thus, we focus on deriving events related to rate changes in transcriptional regulation. A corpus of yeast regulation related abstracts was manually labeled with such events. In order to capture these events automatically, we create an ontology of sub-processes that are likely to result in transcription rate changes by combining textual patterns and biological knowledge. We also propose effective feature extraction methods based on the created ontology to identify the direct evidences with specific details of these events. Our ontologies outperform existing state-of-the-art gene regulation ontologies in the automatic rule learning method applied to our corpus. The proposed deterministic ontology rule-based method can achieve comparable performance to the automatic rule learning method based on decision trees. This demonstrates the effectiveness of our ontology in identifying rate-changing events. We also tested the effectiveness of the proposed feature mining methods on detecting direct evidence of events. Experimental results show that the machine learning method on these features achieves an F1-score of 71.43%.

**Conclusions:**

The manually labeled corpus of events relating to rate changes in transcriptional regulation for yeast is available in https://sites.google.com/site/wentingntu/data. The created ontologies summarized both biological causes of rate changes in transcriptional regulation and corresponding positive and negative textual patterns from the corpus. They are demonstrated to be effective in identifying rate-changing events, which shows the benefits of combining textual patterns and biological knowledge on extracting complex biological events.

## Background

Living cells are the product of gene expression processes which involve regulated transcription over thousands of genes. Transcription is controlled by regulatory proteins binding to specific promoter sequences. The biochemical process by which a collection of regulatory proteins associates with genes across a genome can be described abstractly through a transcriptional regulatory network. There are time delay or lag during the process where the change of expression of the regulator is transmitted to the change in the target gene expression. Gene regulation with time delay is due to the intermediate processes between the product and repressor/inducer. Time delay is thus an important element in the construction of a transcriptional regulatory network [1]. However, the validation of time delay in gene regulation is a challenge as existing biological databases always lack time-related information of gene regulation.

There is an abundance of biological knowledge and facts in scientific articles [2-5]. Due to the ever-increasing corpus of scientific articles, it is difficult, if not impossible, to manually extract these knowledge. Text mining tools can help to automatically extract biological events from literature. For example, a number of relation extraction methods have been applied to extract gene regulation events [6-13].

The simplest approach is the co-occurrence based method [13], which predicts the relation between co-occurring entities at the sentence or abstract resolution. The rationale is that entities that are repeatedly mentioned together are somehow related. Prediction results of the co-occurrence methods typically have a high sensitivity but low specificity. Besides this limitation, details of the regulation such as direction and types cannot be easily determined using this method.

Pattern based extraction methods search for textual patterns of events with the help of predefined rules. Rules can be manually compiled [6] or learned from a training corpora [8,9,14]. To extract and fire a rule, multiple ontologies have to be consulted in addition to bio-entity vocabularies [9,10,14,15]. Concept ontologies, for example, are constructed from text corpora by using formal concept analysis [14]; the BioInfer project provides an ontology for diverse biological events [8,9]; Gene Regulation Ontology [10] is designed to model complex events that are part of the gene regulatory processes. Compared with the co-occurrence based methods, pattern based ones increase the specificity at the expense of a decreasing sensitivity.

Semantics analysis using natural language processing tools can help to extract complex rules from underlying sentences. For example, the RelEx relation extraction system [7] uses a simple set of rules to extract relation paths from dependency parse trees. A set of hand-compiled rules were proposed for linguistic analysis and conceptual inference over semantic structures [12]. The rule-based system then matched syntactic-semantic patterns to the dependency structures. However, extracting rules manually from semantic structures is time-consuming and cannot be done easily on large-scale corpora. A current trend in feature design is to learn the semantic rules automatically via machine learning methods. For example, previous work investigated the performance of different Automatic Content Extraction (ACE) feature sets for biomedical relation extraction in a supervised learning setting [11]. Positive and negative regulation relations in biomedical pathways were learned from the lexical semantic annotations in [16]. Rich graph-based feature sets were proposed to extract complex biological events [17,18].

In this paper, we first construct a corpus by extracting transcriptional regulation rate changing events for yeast from PubMed abstracts. To automatically extract such events, we need to extract the related textual patterns, but there are rarely obvious textual patterns about rate changes in transciptional regulation. We thus create two ontologies from the corpus, based on the biological analysis of sub-processes that may result in rate changes in the transcriptional regulation, and the summarization of the positive/negative textual patterns/rules for describing such sub-processes. We then adopt the effective information retrieval (IR) tools to detect target events. Both automatic rule learning method and simple decision rule based method are used to test the effectiveness of the generated ontologies on identifying rate changes in transcriptional regulation. We also extend the ACE-Style features [11] and graph-based features [17,18] based on our ontologies to obtain a set of conceptual, semantic and sentence graph structure features which can be used to learn the direct evidence for detecting transcriptional regulation rate changing events. The effectiveness of the proposed features has been verified experimentally. A decision tree trained with the proposed features achieves 71.43% F1-score over ten-fold cross-validation.

## Text preprocessing

### Collecting abstracts and trigger words

We downloaded 181,517 abstracts related to yeast from PubMed [19] on September 30, 2012. The downloaded abstracts contain any of the terms "yeast", "Saccharomyces cerevisiae", or "S. cerevisiae" in (i) title, abstract, article fields, or (ii) the head of a Medical Subject Headings (MeSH, a controlled vocabulary for manually annotating PubMed articles) term for the article.

We collected the vocabulary of words related to gene regulations from the BioInfer relation ontology [9] and used heuristics to manually create a list of trigger words relating to rate changes. We then extracted only those sentences from the abstracts that contain both trigger words about gene regulation and rate changes. To find out which trigger words are commonly used in PubMed, we randomly chose hundreds of sentences containing different trigger words and manually pruned them. The trigger word lists were pruned by removing relation words unrelated to regulation and extending the rate change word list with more words.

### Word normalization

To identify all the genes of yeast, we obtained a list of synonyms and identifiers from the Saccharomyces Genome Database [20]. We also manually created a dictionary to cluster semantically similar trigger words together, using a stemming method like in [7]. We then performed word normalization by (i) replacing all gene names with a placeholder word "genename" to eliminate the differences between diverse genes; and (ii) replacing all keywords in the dictionary with their stemmed counterparts, i.e., the words in the first column of the dictionary. Word normalization helps removing the diversity of textual information, especially the article keywords. It is therefore helpful for summarizing textual patterns or rules for events.

### Sentence filter

Since information extraction methods work poorly on irrelevant raw data, we need text filtering to help reduce the variability of textual data. It is commonly assumed that the desired information is local to a sentence [7-9,14]. We thus constrain the event extraction at the sentence resolution.

Using gene regulation and rate change trigger words as filters, relevant sentences were filtered from yeast gene regulation and time related PubMed abstracts. The MEDLINE abstracts were first segmented into individual sentences by using Natural Language Toolkit (NLTK) [21] sentence tokenizer. Afterwards, the sentences are tokenized by NLTK word tokenizer into word tokens. The final set of sentences must satisfy all of the following conditions: (i) there are at least two occurrences of gene/protein names tagged as noun in the sentence; (ii) the sentence matches many words from the "regulate" keyword list; and (iii) the sentence matches many words from the rate change keyword list.

The first filter is easy to implement. To implement the other two filters, we used the cosine similarity to quantify the occurrence of keywords in the sentence. Cosine similarity uses normalized counts that help to remove the bias caused by the overlong sentences. Thus, it can capture more informative sentences than by using occurrence counts. Specifically, each trigger word list (i.e., "regulate" or temporal information) is written as a unit vector y. Each sentence is written as a binary vector × which has a one in the dimension corresponding to a matched word in the sentence from the trigger word list. Vectors × and y have the same dimension as the number of keywords in the trigger list. The cosine similarity of the sentence with the keywords list is then computed as

$$\text{cos}\left(\text{x},\text{y}\right)=\frac{\text{x}\cdot \text{y}}{\left||\text{x}||\;||\text{y}|\right|}$$

where x · y represents the inner product of vectors x and y; and ∥∥ represents the *L*_2 _vector norm.

There are altogether 114,375 sentences that score above zero in both two similarity scores. Since it is infeasible to manually label all of them, we choose only sentences whose cosine similarity to the "regulate" list exceeds 0.1 and whose similarity to the temporal list exceeds 0.15. The final corpus includes 1309 sentences, which are then used as inputs to the next information extraction task.

## Corpus annotation

### Records of transcriptional regulation rate changing event

To the best of our knowledge, we are the first group to extract time-delayed gene regulation evidence from literature. Since specific/quantitative time-related information is not common in literature, much less in abstracts, we detect the events of rate changes in transcriptional regulation instead. The detected rate changes provide qualitative evidence (i.e., shorter or longer) for inferring time delays in transcriptional regulation. Table 1 gives two examples about transcriptional regulation rate changing events. The positive instance shows that "when glucose repression of MTH1 expression is prevented", degradation of MTH1 is slowed, and a delay occurs in "induction of HXT3 expression in response to glucose", thus contains transcriptional regulation rate changing event. As a comparison, the negative instance indicates that "dynamical activities of the key components" undergoes rate changing, but the words "translational control in the expression of cycle proteins Cdc13 and Cdc25" indicates that it is in translational process instead of transciptional process.

**Table 1:** Examples of Transcriptional Regulation Rate Change Events - one positive and one negative.

Negative Instance	As the **translational control in the expression of cycle proteins Cdc13 and Cdc25 **constructs **positive feedback loops**, the **dynamical activities of the key components **undergoes a *rapid rising *after a preliminary stage of *slow increase*. (PMID: 20303984)
Positive Instance	In contrast, **degradation of MTH1 **is *reinforced *by **glucose repression of MTH1 expression**: **disappearance of MTH1 **is *slowed *when **glucose repression of MTH1 expression **is *prevented*, and this results in *a delay *in **induction of HXT3 expression in response to glucose**. (PMID: 16400179)

Words in bold indicate transcriptional regulations while rate changes in the regulation process are italicized.

Note that the rate-changing regulation events are different from positive/negative regulations, which have been studied by others [11,12]. The positive regulation (GO:0010628) and negative regulation (GO:0010629) defined in the Gene Ontology [22] correspond to biological processes that modulate gene expression to increase or decrease, respectively. Both positive and negative regulations belong to gene regulation process. In contrast, the rate-changing regulation studied here refers to a change in the speed or duration of the regulation process. As such, both positive and negative regulations may have rate changes as well.

### Corpus annotation

We annotated the corpus by manually labeling sentences containing transcriptional regulation rate changing events as positive instances and others as negative instances. For positive instances, we identified trigger words that indicate mentions of transcriptional regulation processes or rate changes of the processes. These words were annotated to facilitate the creation of our time-delay (transcriptional regulation rate change) ontology. In the negative class, the sentence may only include information about gene regulation without rate changes or about a biological process other than transcriptional regulation.

Both direct and indirect evidences exist in the positive instances. We thus further annotate the positive class with two types of events: (i) events with specific information about regulator, regulatee and rate changes in transcription regulation, and (ii) indirect evidences for transcription regulation rate changing events.

A biological practitioner was employed to annotate the corpus. Since there is a single annotator, we are unable to report the inter-annotator agreement score about our annotation. However, during the ontology extraction process, whenever we found annotations inconsistent with the extracted ontologies (biology knowledge), the annotator was asked to double check and correct all annotations (especially the positive class) again.

## Biological knowledge driven ontologies for rate changes in transcriptional regulation events

**Figure 1:**
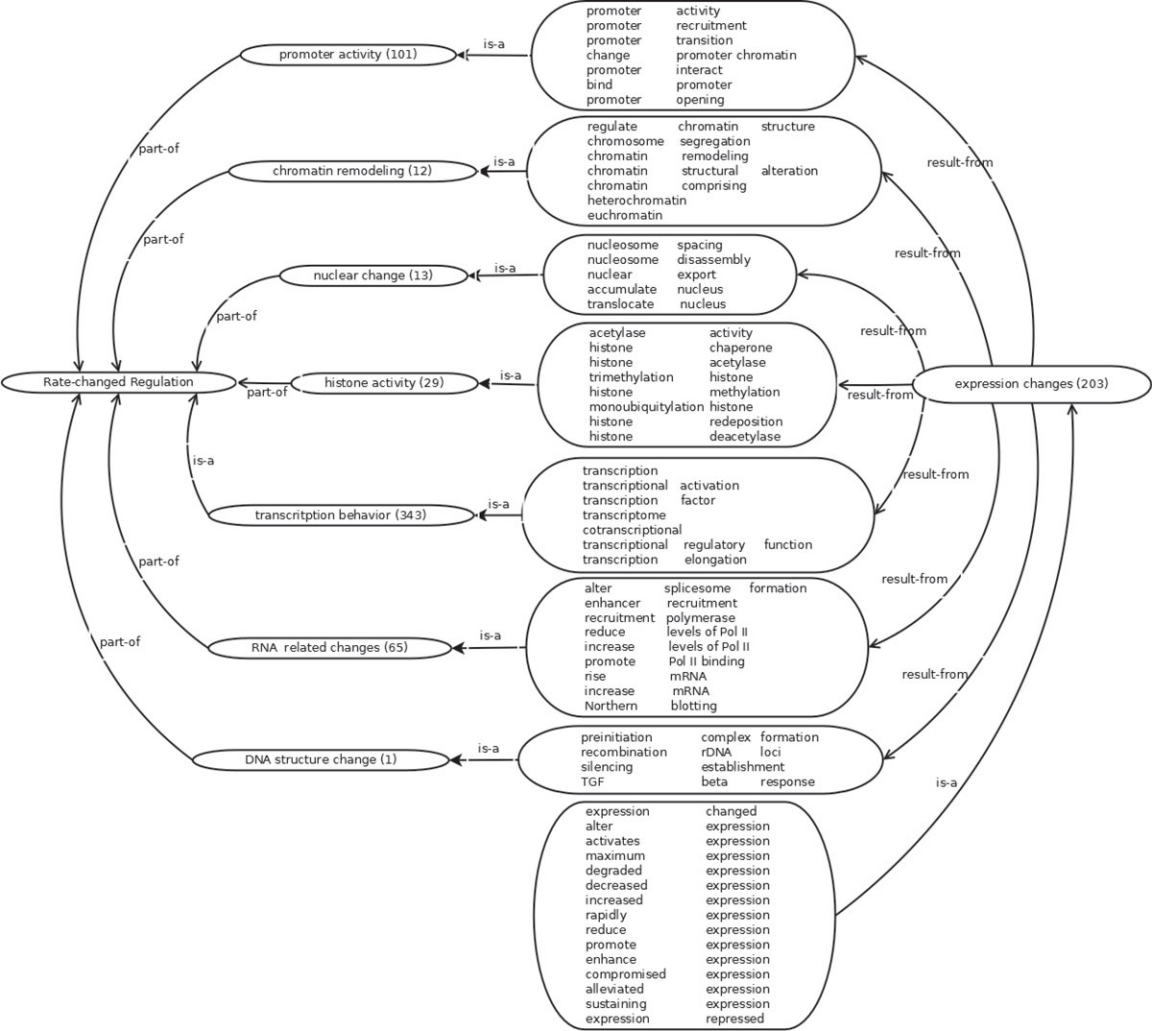
**Transcriptional Regulation Rate Change Ontology**. The column of big boxes contain the textual patterns corresponding to the sub-processes of transcriptional regulation rate change events. The smaller boxes contain processes or classes of events. The numbers of instances (sentences) corresponding to the process in the corpus are indicated in the bracket next to each process.

**Figure 2:**
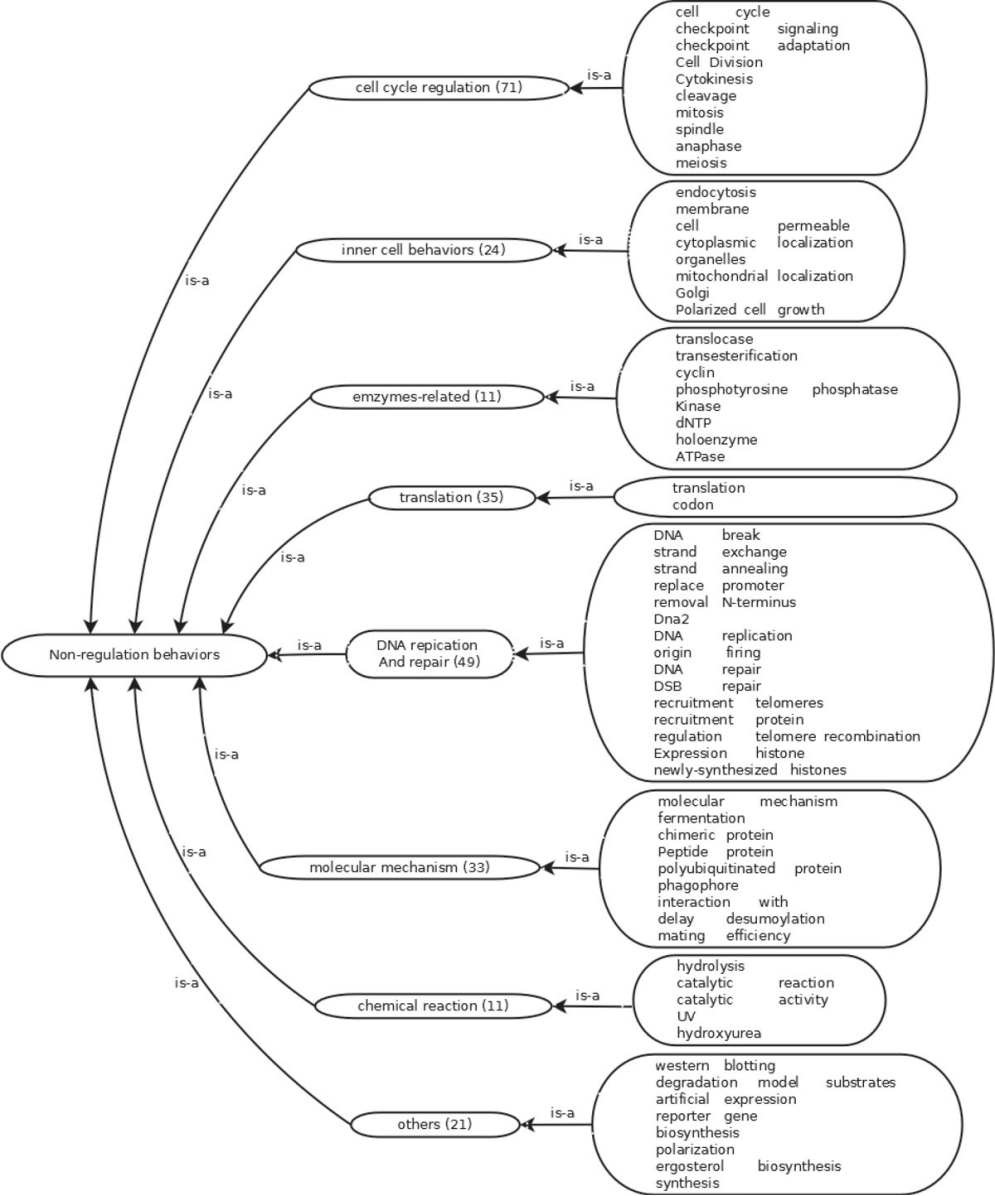
**Negative Transcriptional Regulation Ontology**. The rightmost column of big boxes contain textual patterns corresponding to the sub-processes of negative instances. The other boxes contain processes or classes of events. The numbers of instances (sentences) corresponding to the process in the corpus are indicated in the bracket next to each process.

By combining statistical analysis on the annotated trigger words with biological knowledge on gene regulation, we were able to create two ontologies. The first ontology, as shown in Figure 1, summarizes the textual patterns and biological reasons for rate changes in transcriptional regulation. The second ontology summarizes the negative rules as illustrated in Figure 2.

In order to make our ontologies consistent with previous work, we also provided logical relations between subclasses, events, or processes. Following the convention used in the Gene Ontology database, *is *− *a *represents the subtype relation and *part *− *of *is part-whole relation. Following the Gene Regulation Ontology [10], *result *− *from *refers to the result of some processes or explanations. We also list transitive rules among these relations, which is consistent with the Gene Ontology relation [23] and Gene Regulation Ontology as follows.

∀ relation *r *∈ {*is *− *a*, *part *− *of *, *result *− *from*}, we have rules *r *○ *is *− *a *→ *r *and *is *− *a *○ *r *→ *r*; moreover,

$$\begin{array}{c} part-of\circ result-from\to result-from\\ result-from\circ part-of\to result-from\\ part-of\circ part-of\to part-of \end{array}$$

where *r*_1 _○ *r*_2 _→ *r*_3 _means if A has relation *r*_1 _with B, and B has relation *r*_2 _with C, we can infer that A has relation *r*_3 _with C. Thus, according to these transitive rules, we infer more relations between two terms if an inferred relation path exists between them according to the method in [24].

### Biological knowledge based analysis about rate changes in transcription regulation

The stages in which gene expression is regulated typically include: (i) Chromatin domains; (ii) Transcription; (iii) Post-transcription; (iv) Translation; and (v) Post-translation. The first two stages are mostly related to transcriptional regulation. We thus analyze the causes in these processes that may result in transcriptional regulation rate change. The textual patterns for transcriptional regulation rate changes related to these two processes are as follows.

(i) Chromatin domains. Unlike prokaryotic, eukaryotic linear DNA moleculars coil with nuleosomes tightly and package into choromosomes. When some genes are to be expressed, the region of the activation zone is unpacked, the nuleosome is decomposed, and then transcription related proteins come inside to form transcription complex and perform transcription initialization. Choromosome unpacking is often related to several events such as nuleosomes composition and decomposition, and chromosome structure modification. Thus, all of these events are the sub-events that result in transcriptional regulation rate changes. Some chemical modifications around histones may change the chromosome structure, which can eventually affect transcription [25]. The processes that modify histones include DNA methylation, microRNA, or DNA-binding of proteins. Example-1 in Table 2 indicates that gene "Mdm2" ubiquitylates chromatin proteins around the area of target promoter and controls the transcription level of the target gene. The Example-2 in Tabel 2 indicates that gene "chz1" relates to the ubiquitination of histone H2B and di-methylation. As a result, the rate of binding process of "Sir3p, and Sir4p" is increased.

**Table 2:** Transcriptional Regulation Rate Change Pattern Examples.

Example-1	Endogenous Mdm2 is tethered in vivo, presumably via p53, to **chromatin comprising **the p53-responsive p21(waf1) promoter, and Mdm2 overexpression *enhances ***protein ubiquitylation **in the vicinity of a p53 binding site within that promoter. (PMID: 15546622)
Example-2	Deletion of CHZ1 led to *reduced ***ubiquitination **of subtelomere-associated H2B, *reduced *subtelomeric H3K79 **dimethylation**, and *increased *binding of Sir3p, and Sir4p at telomere-distal **euchromatin **regions, correlating with *decreased ***gene expression **in subtelomeric regions. (PMID: 20008511)
Example-3	The cofactor npl4-1 and ufd1-2 mutants also exhibit G1 delay and *reduced *CLN1 **promoter activity **at 38*:*5°C, suggesting that Npl4-Ufd1 complex mediates the function of Cdc48 at G1. (PMID: 21526151)

(ii) Transcription. Transcriptional regulation controls when transcription happens and how many copies of RNA are synthesized. Transcription factors help RNA polymerase bind to the target region. In this process, protein-DNA and protein-protein interactions are involved. Factors that affect the function of transcriptional factors or RNA polymerase eventually lead to transcriptional regulation rate changes, e.g., promoter activity, RNA polymerase activity, and RNA polymerase binding event. Example-3 in Table 2 indicates that cofactor genes np14-1 and ufd1-2 are related to the expression of gene cln1 by affecting the activity of the cln1 promoter.

### Negative rules for transcriptional regulation

We have applied the transcription rate change ontology on the corpus with the goal of predicting transcription regulation rate changing events. We obtained satisfactory recall value. But there were many false positive counts. By analyzing false positive samples, we discovered some trigger words in the ontology, such as regulation, binding, reducing, and delay, which are widespread in many biological processes, but are not related to transcription. We thus generated the negative transcriptional regulation ontology based on the analysis of trigger words in the false positive sentences. The negative rules for transcriptional regulation are shown in Figure 2. They are primarily derived from three types of events, namely the cell cycle regulation, DNA replication, and regulation of other biological processes.

Cell cycle is the process of cell-division, which is sophisticatedly regulated by a series of cell signaling events and gene regulation. Thus, when we apply "regulate" class of rules, we need to combine it with cell cycle negative rules. Example-4 in Table 3 contains several keywords associated with activation but its target is not transcription. The regulation here refers to the cell-cycle as indicated by the terms "nuclear segregation" and "anaphase I spindle".

In DNA replication processes, chromosomes must be decomposed and nucleosomes need to re-deposited to form newly-synthesized histones. Thus, when we use rules of histone and chromosomes behaviors, we consider DNA replication based negative rules. Example-5 in Table 3 includes keywords about chromosome behaviors such as "chromatin-remodeling"; however, these behaviors actually relate to DNA replication instead of gene expression. The keyword "replication fork" positively confirms this to be a process of DNA replication.

**Table 3:** Negative Patterns Examples.

Example-4	We show here that the role of these proteins is instead to promote nucleolar segregation, including release of the Cdc14 phosphatase required for Cdk1 inactivation and disassembly of the anaphase I spindle. (PMID: 12737807)
Example-5	Here we show that two highly conserved ATP-dependent chromatin-remodeling complexes in Saccharomyces cerevisiae, Isw2 and Ino80, function in parallel to promote replication fork progression. (PMID: 18408730)
Example-6	Of the single codon changes, mutation of the first ATG (ATG1) resulted in the largest increase of the reporter gene PIS1(promoter)-lacZ expression. (PMID: 16997274)

Specific biological processes other than transcription are also included in the negative rules. Example-6 in Table 3 contains the term "codon", which indicates it to be translation process.

The negative transcriptional regulation ontology helps filter out events that are textually similar to transcription regulation events. Note that each sentence in our corpus contains both trigger words for regulation and rate changes. Hence, the negative transcriptional regulation ontology is based on the analysis of sentences that have similar textual patterns with rate-changing regulation events but are in fact not. They can help reduce the number of false positives from using the regulation ontology alone.

### Statistical analysis on ontologies

**Figure 3:**
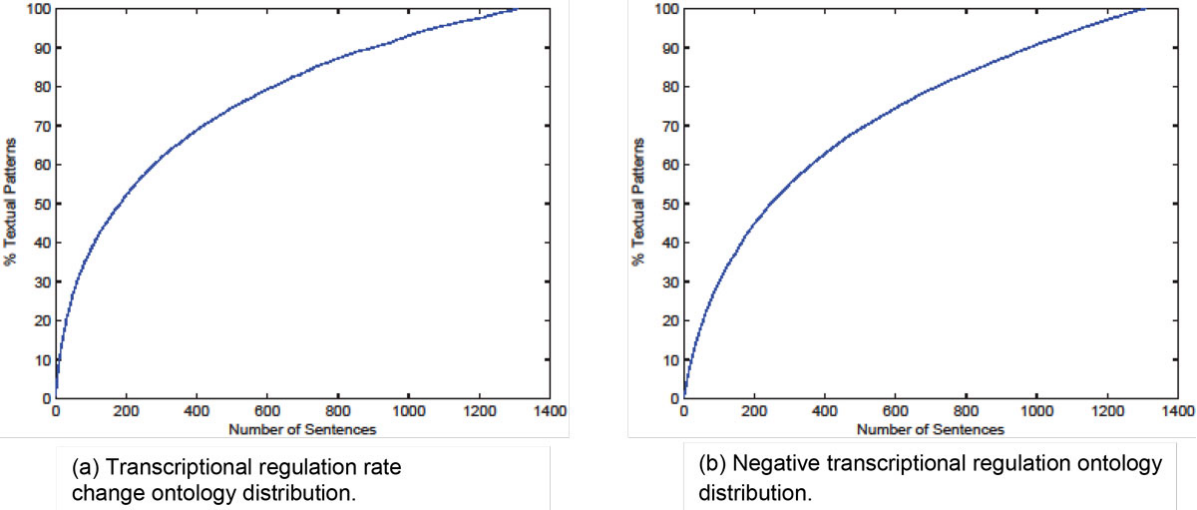
**The average cumulative distribution of textual patterns from each ontology, over the number of sentences**. The data is taken from the average over 100 random shuffles of the corpus sentences.

To test the coverage of the textual patterns embedded in both ontologies, we shuffle the sentences and plot the average cumulative fraction of textual patterns that occur in randomly ordered sentences in Figure 3. The figure shows that the first 20% randomly selected sentences contain 60% of the transcriptional regulation rate change rules embedded in the regulation ontology, and 53% negative rules listed in the negative ontology. This demonstrates that our ontologies have a good coverage.

Figures 1 and 2 also list the number of matching sentences in each subclass of the two ontologies. For example, the two largest counts in the rate change ontology come from promoter activity (101) and transcription behavior (343). Some sub-classes have just a handful of instances, such as nuclear change (13), chromatin remodeling (12), DNA structure changes (1), chemical reaction (11). However, due to their important biological implication on the transcriptional regulation or negative events, we also include them into the ontology. This makes our ontology biologically more feasible and complete.

A large number of sentences contain process descriptions that resemble regulation but are in fact non-transcription regulations. This can be seen in the large counts of non-regulation processes. Keywords such as "regulate", which are used in transcription regulation, are in fact quite generic, i.e., they are used to describe other regulation processes like cell cycle regulation (71), translation (35), DNA replication and repair (49). This demonstrates the necessity of having a negative ontology of rules.

## Identifying transcriptional regulation rate changing events with rule-based methods

### evaluating ontologies by automatic rules learning method

We compared the proposed ontologies with two benchmarks: BioInfer ontology [9] and GeneReg ontology [10]. The former has been used to recognize diverse biological events [8,9]. The latter is designed to model complex events that are part of the gene regulatory processes [10]. We extracted gene regulation rules from the BioInfer ontology. We then extracted trigger words from both ontologies as our regulation rules. To ensure a fair comparison, we extracted trigger words indicating rate changes from our ontologies. The extracted trigger words are listed in Table 4.

**Table 4:** Rate Change Trigger Words.

Quicken	activate, accelerate, exert, extend, increase, rise, peak, drastically, rapidly, positive, promote, enhance, assist, acetylase inhibit
Delay	block, deacetylase, prevent, suppress, abolish, reduce, decrease, degrade, compromise, sustain, alleviate, decline, lower, negative, repress, diminish, limit, fail, lack, delay, late, slow, shut down, less effect, turn off
Change	rate change, affect rate, alter, over period, during period

We used the decision tree from the Weka machine learning package [26] as a tool to evaluate the discriminative power of these ontologies on identifying the transcriptional regulation rate changing event. Specifically, we built several decision trees, each of which uses a specific ontology-based rules as rule bases (features). For the BioInfor ontology and the GeneReg ontology, we incorporated them with rate change trigger words as the rule (feature) set. For the proposed regulation rate change ontology and negative rules, besides testing them individually, we also evaluated a combination. These decision trees are trained on a subset of the aforementioned corpus. They actually act as an automatic rule pruning process [27]. After training, the refined rules are tested over another portion of the corpus. By comparing the testing results, we see the discriminative power of various ontologies.

Table 5 reports ten-fold cross-validation results of training decision trees with rule sets derived from various ontologies. It can be seen that our ontologies outperform the two benchmarks on all metrics. The proposed regulation rate change ontology can predict the positive class with high precision and recall. The proposed negative rules can help reduce the false positive rate and improve precision of regulation rate change ontology; the combined ontologies thus achieve the highest F1-score of about 76%. This demonstrates the effectiveness of both our regulation rate change ontology and negative rules.

**Table 5:** Performance of decision tree prediction using rule sets derived from different ontologies.

Model	*TP *	*FP *	*FN *	*TN *	*Prec.(%) *	*Rec.(%) *	*F1(%) *	*Acc.(%) *
BioInfer ontology	223	121	134	831	64.83	62.46	63.62	80.52

GeneReg ontology	235	109	122	843	68.31	65.83	67.05	82.35

Regulation rate change ontology	**276**	107	81	845	72.06	**77.31**	74.59	85.64

Combined ontologies	**277**	**99**	80	853	**73.67**	**77.59**	**75.58**	**86.33**

### Deterministic rule-based methods

The proposed ontologies can be used to derive rules for identifying transcriptional regulation rate changing events. A straightforward rule is to treat sentences in the corpus as a positive instances if they contain sub-events from the regulation rate change ontology. We name this as "Regulation-based Rule". Table 6 shows the results of predicting transcriptional regulation rate changing events using this method. Venturing a step further, we also explored the feasibility of combining both the regulation rate change ontology and negative rules based ontology. Specifically, the following rules are used to predict transcriptional regulation rate changing events.

**Table 6:** Classification performance of various ontology rule-based methods.

	*TP*	*FP*	*FN*	*TN*	*Prec.(%)*	*Rec.(%)*	*F1(%)*	*Acc.(%)*
Regulation-based Rule	335	223	22	729	60.04	**93.84**	73.22	81.28

Combined Rule	322	**158**	35	**794**	**67.08**	90.20	**76.94**	85.26

• If a sentence only contains sub-events from the regulation rate change ontology, it is classified as a positive instance.

• If it also contains sub-events from the non-regulation ontology, in addition to containing sub-events from the regulation rate change ontology, we classify it as a negative instance.

• If no sub-events from the regulation rate change ontology exist in the sentence, it is classified as a negative instance.

The performance of the "Combined Rule" approach is shown in Table 6.

From Table 6, it can be seen that both methods are able to identify transcriptional regulation rate changing events with reasonable accuracies. By comparing false positive (FP) counts of the "Regulation-based Rule" with the "Combined Rule", we see that non-regulation ontology has successfully reduced the false positive counts from 223 to 158. Both precision and F1-scores are improved, with a slight degradation (3%) in recall. We also notice that the performance of our deterministic rule-based methods are comparable to that of the decision tree approach using "Combined ontologies" as shown in Table 5. This demonstrates the effectiveness of our ontology on discriminating transcription regulation rate changing events from other regulation events.

## Identifying direct evidences for events by machine learning methods

The above rule-based methods are capable of identifying transcriptional regulation rate changing events, but fail to identify specific details of the rate-changing regulation events. The details are important as they are indispensable for extracting gene regulatory network (GRN) from literature. To extract detailed information, we need to consider the position of genes and their relations such as part-of-speech (POS) tags and grammatical relations between keywords, etc.

### Direct and indirect evidence records for events

Through manual inspection of the transcription regulation rate changing events, we discovered that there are direct or indirect evidences of transcription regulation rate changing events. The direct evidences provide complete information about the regulator, regulatee and rate changes of the event. The indirect evidences in the positive class have following two subclasses:

1 Subclass I: incomplete regulation information. The sentence provides incomplete or partial regulation information. For example, according to the snippet "a decrease in the amount of HSP12 and HSP104 mRNAs" of Example-7 in Table 7, we know that the expression of "HSP12 and HSP104" changes. However, we are not sure about the regulation relationship and regulation rate change. Thus, we need more information to confirm it as a regulation rate changing event.

**Table 7:** Examples of indirect evidence.

Example-7	The results indicate that during the first hours of microvinification there is an increase in the GPDI mRNA levels with a maximum about one hour after inoculation, and a decrease in the amount of HSP12 and HSP104 mRNAs, although with differences between them. (PMID: 12086182)
Example-8	Four different conditions were found to cause expression of Ime1 protein in vegetative cultures: elevated transcription levels due to the presence of IME1 on a multicopy plasmid; elevated transcription provided by a Gal-IME1 construct; G1 arrest due to alpha-factor treatment; G1 arrest following mild heat-shock treatment of cdc28 diploids. (PMID: 8483452)

2 Subclass II: genetic engineering manipulation. The sentence may include information about gene regulation and transcription that are unnatural, which means that the gene information included in the sentences are not necessary the original genes in our target organism. As Example-8 shown in Table 7, "multicopy plasmid" and "GalIME1 construct" indicates that the related genes are external or went through some engineering manipulation. Thus, this type of events only provide indirect evidence for our target event; more information is needed to confirm that the genes are indeed original.

To extract detailed information about transcriptional regulation rate changing events, we need to detect direct evidence from indirect evidence. As indicated by the examples shown in Table 7, direct and indirect evidence have similar textual patterns. We thus need to formulate semantic structural features to extract the direct evidence.

### Learning direct evidences by decision tree

We propose a feature-based method that incorporates diverse lexical, syntactic and semantic features to automatically extract transcriptional regulation relations. Inspired by the Automatic Content Extraction (ACE) program features [11] and state-of-the-art rich graph features [17,18], which have proven useful in detecting relations between entities, we define three classes of features, bag-of-words, graph and shortest path features as follows:

1 Keyword-tag: a combination of the keywords defined in our ontologies, and their POS tags, which indicate their grammatical roles in sentences. The keywords in the features are normalized to reduce the diversity of words with the same tags.

2 Word-relation-word: two words concatenated by the name of their dependency relation type. The relation is extracted from the shortest relation path between genes and keywords in the dependency tree derived from the Stanford NLP parser [28].

3 Gene-keyword-distance: a triplet of gene, keyword, and length of the shortest relation path between them in the dependency tree.

Compared with the classical window scoping and chunking entity recognition approach [11], we extract more concise and informative word features (keywords) by using our structured ontologies. We also consider the tag types of keywords in addition to considering the entity type [11]. In [11], the full parsed features include the path of phrase and phrase labels between two entity mentions in the parser tree. To consider relational trigger words, they also exploit features indicating whether the top phrase in the parse path between the entity mentioned contains the relation triggers. We also consider these two kinds of features, but express them differently with the help of the informative trigger words. Similar to the full parsed features in [11], we consider the dependence relations between entity genes and the relation trigger words. Specifically, we denote the shortest path in the full parse tree between them as the "word-relation-word" form. Thus, all phrase labels are in fact shown independently and the phrase words are also included. We summarize the shortest path length between gene and keywords as the "gene-keyword-distance" feature. Hence, we in fact re-formalize and improve the ACE features in [11] to use our ontology for improved automatic relation extraction.

With the help of the informative keywords provided by our ontologies, the proposed features actually incorporate the state-of-the-art rich graph features [17,18]. Specifically, "keyword-tag" feature is the combination of "token features" and "POS of tokens" in [17,18]. Instead of using all tokens in the path, we choose more informative keywords from our ontologies. The "word-relation-word" feature combines the 2-gram consecutive tokens and their "edge feature" (i.e., dependency type of the edge) in the path [17,18]. And "gene-keyword-distance" feature covers the "frequency feature" which is the length of the shortest path [17,18]. Thus, the proposed features in fact cover state-of-the-art features [17,18] and are more informative due to our ontologies. In fact, we also implemented the experiment that adding all tokens in the path and their POS into our features, but it didn't improve any performance. This indicates that the keywords from our ontologies provide enough information of tokens.

After extracting the graph features, we perform following post-processing to reduce the diversity of features: (i) for the dependency type of edge in the path, we use generic types (i.e., the dependency types in the second level of hierarchy tree of typed dependencies as shown in [29]) instead of more specific ones; and (ii) for the length of the shortest path, we use three nominal features (i.e., near, moderate and far) instead of the specific value. If the length is less than three, it is treated as "near"; if the length ranges from four to six, it is treated as "moderate"; and if the length is larger than six, it is treated as "far". Moreover, to reduce the word diversity in the features, we also performed word normalization by using their normalized representation instead of original forms in texts. Experimental results show that applying post-processing on extracted features can improve the performance.

There are 357 sentences in total, where 211 sentences belong to direct evidence classes, with the remaining belonging to indirect evidence classes. To evaluate the proposed feature-based approach, we performed tenfold cross-validation on the rate-changing transcriptional regulation events by using the decision tree as the classification method. The results are reported in Table 8 with the row header "Combined Features".

**Table 8:** Performance of ten-fold cross-validation decision tree methods with various feature sets on identifying direct evidence instances.

	*TP*	*FP*	*FN*	*TN*	*Prec.(%)*	*Rec.(%)*	*F1(%)*	*Acc.(%)*
Baseline features [11]	131	88	80	58	59.82	62.09	60.93	52.94

All rules features	149	102	62	44	59.36	70.62	64.50	54.06

Original combined features	175	121	36	25	59.12	82.94	69.03	56.02

Combined features	185	122	26	24	60.26	**87.68**	**71.43**	58.54

As a baseline, we implemented the ACE features as described in [11]. They use all words between entities/keywords mentions as word features. Parser related features among entities/keywords are also included. The results of this method are shown in Table 8 with the row header "Baseline features". To show the performance of ontologies on identifying direct evidences, we treated all textual patterns from the two ontologies as features. The results are listed in Table 8 with the row header "All rules features". To verity the effectiveness of post-processing, we implemented the experiment on combined features without applying post-processing. The results are shown in Table 8 with the row header "Original combined features", which also indicate the performance of using graph features in [17,18]. Benchmarking against these baselines is important as it shows whether the proposed features are more effective than baseline features [11], and in fact necessary to identify direct evidences.

As shown in Table 8, the proposed "Combined features" significantly improve the recall (about 26% improvement) compared to the baseline. It demonstrates the importance of incorporating semantic patterns in the features. Besides, the proposed features achieved an F1-score of 71.43%, which is about 11% better than the baseline. This shows that the proposed features are effective in mining patterns from direct evidence instances.

When direct evidence records are detected, the specific information of rate-changing transcriptional events are identified automatically from the regulation, gene/protein name, and rate change related trigger words. The rate change directions are intuitively identified from the class of rate changes related trigger words as shown in Table 4. As suggested in [11], we can use the 13 frequent textual patterns between "agent" and "patient" to identify regulator and regulatee from gene/protein names. Besides, the effective sentence simplification rules described in [30] can be adopted to remove the irrelevant information, i.e. the noisy genes or keywords.

## Conclusions

In this paper, we manually created a corpus containing events of rate-changing transcriptional regulation, which can be downloaded from https://sites.google.com/site/wentingntu/data. By statistically analyzing the textual patterns of positive instances, combined with biological reasoning over the transcriptional regulation rate change, we cataloged sub-processes and their textual patterns into a transcriptional regulation rate change ontology. Similarly, we established an ontology to collect negative transcriptional regulation textual patterns. Experimental results show that our ontologies outperform state-of-the-art gene regulation ontologies when used together with our decision tree based rule learning method on our corpus. We also proposed some deterministic decision rules by using two established ontologies to identify the events. Experimental results show that this deterministic rule-based method can achieve comparable performance with the automatic rule learning method. This demonstrates the effectiveness of our ontologies and deterministic decision rules on identifying the transcriptional regulation rate changing events.

Since both direct and indirect evidences exist in the transcriptional regulation rate changing events, we need to figure out direct evidence to confirm the final transcriptional regulatory network with rate changes. However, ontology based rules fail to identify direct evidence due to the similarity of their textual patterns with indirect evidence. We thus proposed effective feature extraction methods based on the ontologies to identify direct evidence of events. Experimental results show that it achieves a 71.43% F1-score for ten-fold cross-validation. This demonstrates the effectiveness of the proposed feature mining methods to identify direct evidences.

The detected transcriptional regulation rate changing events can be used as a guidance on detecting time delays from gene expression data [31]. It is also easy to use our corpus and ontologies to collect other related events. As our ontology catalogs sub-events of transcriptional regulation rate changing events, it can be applied for sub-events detection, e.g., promoter activity, expression changing events, etc. How to combine the context information to help predict the event information from the indirect evidence remains an open problem.

## Competing interests

The authors declare that they have no competing interests.

## Authors' contributions

WL developed and implemented the method. KM annotated the corpus and helped extract the ontologies. GL contributed to the processing of full-text articles. WL wrote the manuscript. KC and JCR helped revise the manuscript. GL and JZ gave feedback to the manuscript. All authors have read and approved the manuscript.

## Declarations

The publication costs for this article were funded by Singapore's Ministry of Education Academic Research Tier 2 fund MOE2010-T2-1-056 and Tier 2 grant of ARC9/11.

This article has been published as part of *BMC Bioinformatics *Volume 15 Supplement 2, 2014: Selected articles from the Twelfth Asia Pacific Bioinformatics Conference (APBC 2014): Bioinformatics. The full contents of the supplement are available online at http://www.biomedcentral.com/bmcbioinformatics/supplements/15/S2.
